# Clonal evolution and antigen recognition of anti-nuclear antibodies in acute systemic lupus erythematosus

**DOI:** 10.1038/s41598-017-16681-y

**Published:** 2017-11-27

**Authors:** Shuhei Sakakibara, Takao Arimori, Kazuo Yamashita, Hideyuki Jinzai, Daisuke Motooka, Shota Nakamura, Songling Li, Kazuya Takeda, Jun Katayama, Marwa Ali El Hussien, Masashi Narazaki, Toshio Tanaka, Daron M. Standley, Junichi Takagi, Hitoshi Kikutani

**Affiliations:** 1 Laboratory of Immune Regulation, Immunology Frontier Research Center, Osaka University, Suita, Osaka, 565-0871 Japan; 2 Laboratory of Protein Synthesis and Expression, Institute for Protein Research, Osaka University, Suita, Osaka, 565-0871 Japan; 3 Laboratory of Systems Immunology, Immunology Frontier Research Center, Osaka University, Suita, Osaka, 565-0871 Japan; 4 Graduate School of Medicine, Osaka University, Suita, Osaka, 565-0871 Japan; 5 Department of Infection Metagenomics, Genome Information Research Center, Research Institute for Microbial Diseases, Osaka University, Suita, Osaka, 565-0871 Japan; 6 Laboratory of Immunopathology, Immunology Frontier Research Center, Osaka University, Suita, Osaka, 565-0871 Japan; 7 Department of Respiratory Medicine and Clinical Immunology, Graduate School of Medicine, Osaka University, Suita, Osaka, 565-0871 Japan; 8 Department of Clinical Application of Biologics, Graduate School of Medicine, Osaka University, Suita, Osaka, 565-0871 Japan; 9 Laboratory of Integrated Biological Information, Institute for Virus Research, Kyoto University, Kyoto, Kyoto, 606-8507 Japan

## Abstract

The evolutional process of disease-associated autoantibodies in systemic lupus erythematosus (SLE) remains to be established. Here we show intraclonal diversification and affinity maturation of anti-nuclear antibody (ANA)-producing B cells in SLE. We identified a panel of monoclonal ANAs recognizing nuclear antigens, such as double-stranded DNA (dsDNA) and ribonucleoproteins (RNPs) from acute SLE subjects. These ANAs had relatively few, but nonetheless critical mutations. High-throughput immunoglobulin sequencing of blood lymphocytes disclosed the existence of sizable ANA lineages shearing critical mutations intraclonally. We further focused on anti-DNA antibodies, which are capable to bind to both single-stranded (ss) and dsDNA at high affinity. Crystal structure and biochemical analysis confirmed a direct role of the mutations in the acquisition of DNA reactivity and also revealed that these anti-DNA antibodies recognized an unpaired region within DNA duplex. Our study unveils the unique properties of high-affinity anti-DNA antibodies that are generated through antigen-driven affinity maturation in acute phase of SLE.

## Introduction

Systemic lupus erythematosus (SLE) is a chronic autoimmune disease affecting skin, kidneys and other organs^1,2^. The sera of SLE patients contain high titers of anti-nuclear antibodies (ANAs), which are reactive to nuclear components such as DNA, histones, and heterogeneous nuclear ribonucleoproteins/Smith antigen (RNP/Sm)^1^.

The generation process of ANAs has been studied using SLE mouse models^3^. A number of studies on monoclonal antibodies (mAbs) from these mice have revealed several characteristics of autoantibodies including a crucial role of somatic hypermutation (SHM)^4–7^. On the other hand, studies of monoclonal ANAs derived from SLE patients are limited. Several human ANAs have been shown to react with nuclear antigens in a SHM-dependent manner^8–10^ as reported for mouse ANAs. However, some reports have shown that ANAs can be generated without somatic mutation and rigorous affinity maturation^11–13^. Thus, it still remains unclear how much antigen-driven affinity maturation contribute to the generation of ANAs in human SLE patients. This is largely due to a limited number of *bona fide* human ANA clones and a lack of detailed phylogenetic analysis of disease-associated ANA clones.

Among various ANAs, anti-double-stranded DNA (dsDNA) antibodies are a reliable diagnostic marker for SLE. Previous genetic analysis of murine monoclonal anti-dsDNA antibodies revealed a high frequency of basic amino acids in the complementarity-determining regions (CDRs), inferring that they contribute to electrostatic interactions with the DNA backbone^5^. A hypothetical structural model of anti-dsDNA antibody has demonstrated that the tips of the heavy chain CDR1 and 2 of (HCDR1 and 2) extend into the major groove of the dsDNA allowing HCDR3 to make contact with the phosphate backbone^5^. A similar model for human anti-dsDNA antibody has also been reported^14^. Yet, these models have not been validated by crystallographic analysis.

In the current study, we characterized disease-associated ANAs isolated from SLE patients in the acute phase. High-throughput sequencing (HTS) analysis was performed to understand the evolutionary process of the ANAs. Furthermore, we performed *in silico* docking and x-ray crystallography on a representative anti-dsDNA antibody, which revealed a novel structural basis of antigen recognition by anti-DNA antibodies.

## Results

### Circulating CD138^+^ cells represent serological anti-nuclear reactivity in the acute phase of SLE

**Figure 1:**
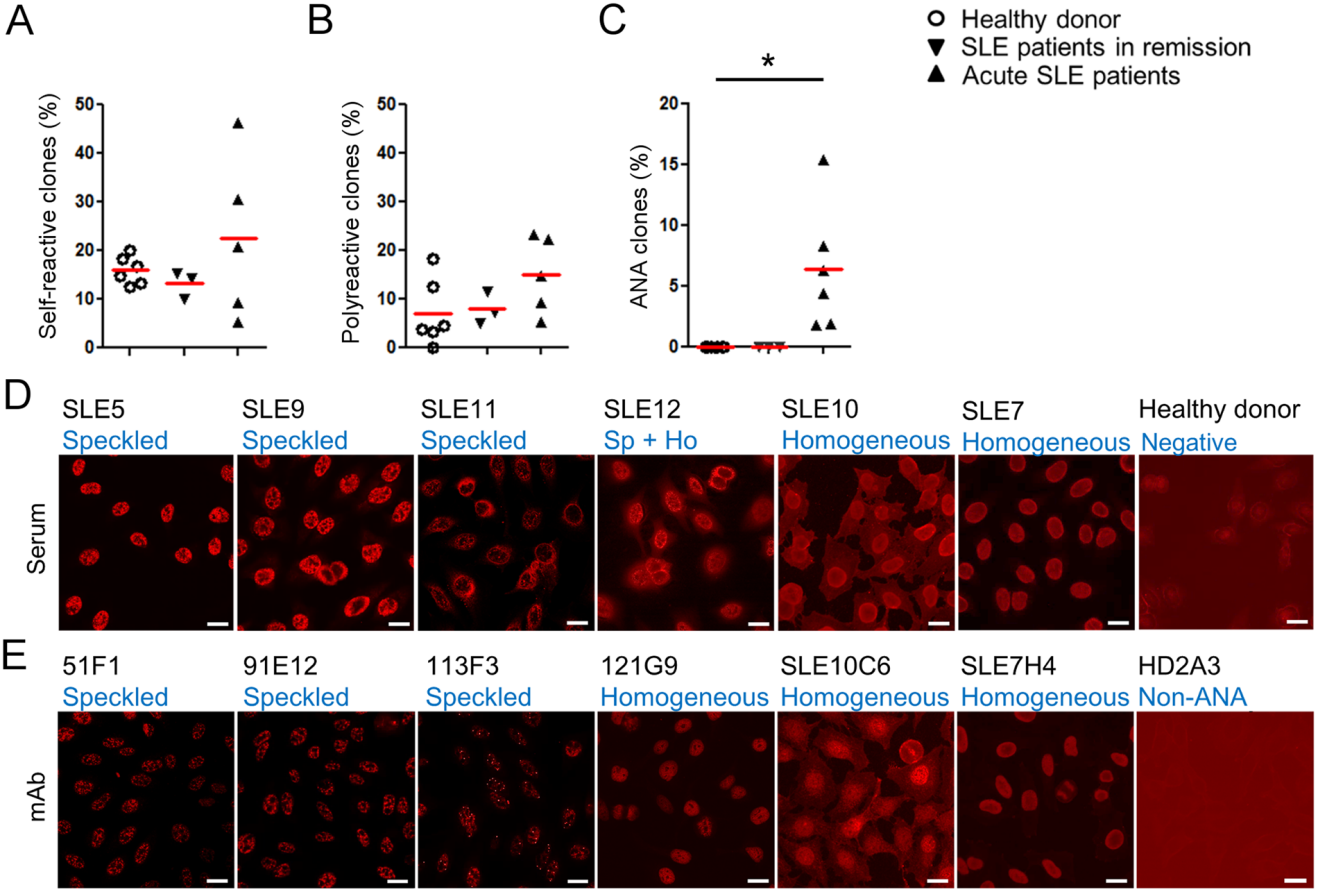
Isolation of disease-associated autoantibody clones in SLE. (**A**–**C**) Summary of self-reactive tests for reconstituted monoclonal antibodies. Percentages of self-reactive clones determined by anti-dsDNA and anti-CL ELISA (**A**), polyreactive clones to self-antigens (dsDNA, CL and insulin as a unrelated self-antigen) (healthy donors [6 donors, n = 150], SLE patients in remission [3 donors, n = 74], and SLE acute patients [5 donors, n = 199]) (**B**), and ANA clones in IFA (healthy donors [6 donors, n = 150], SLE patients in remission [3 donors, n = 74], SLE acute patients [6 donors, n = 239]) (**C**). (**D** and **E**) IFA. Hep 2 cells were stained with diluted sera (**D**), dilution factor = 1:160 or more for acute SLE subjects, 1:40 for healthy control), or represented monoclonal ANA clones (2 μg/ml for SLE10C6; 0.67 μg/ml for 51F1, 91E12, and 113F3; 0.2 μg/ml for 121G9 and SLE7H4), or non-ANA clone HD2A3 (2 μg/ml). Bars = 20 μm (**E**).

To isolate disease-associated autoantibodies, we generated 199, 74, and 150 mAb clones from blood CD19^low^ CD138^+^ plasmablasts (PBs) of untreated acute SLE patients, patients in remission, and healthy volunteers, respectively (Supplementary Fig. 1 and Supplementary Table 1), and tested for their reactivity against representative SLE self-antigens, dsDNA and cardiolipin (CL) in ELISA. We observed no difference in the frequencies of autoantibodies against dsDNA or CL among the three groups (Fig. 1A). Most of such autoantibodies were polyreactive, even reactive to insulin which is unrelated to SLE. Thus, unexpectedly high frequencies of self-reactive and polyreactive clones were comparably observed in PBs of healthy donors, SLE patients in remission and patients in the acute phase (Fig. 1B). On the contrary, in indirect immunofluorescent staining assay (IFA) with Hep2 cells, 14 out of 239 clones from 6 acute subjects showed strong nuclear reactivity at concentrations lower than 2 μg/ml (Table 1 and Supplementary Table 2). The isolated ANA clones represented ~6% of the PB-derived mAbs isolated from acute patients, but not from patients in remission or healthy donors (Fig. 1C), which was consistent with serum ANA titers of our subjects (Supplementary Fig. 1). Furthermore, these ANAs showed nuclear staining patterns that recapitulated those with the respective donors’ sera. Three ANAs from donor SLE5 and serum from this patient exhibited similar speckled staining patterns (Fig. 1D,E and Supplementary Fig. 1). Most ANA clones from patient SLE7 reproduced homogeneous staining patterns of the donor’s serum (Fig. 1D,E and Supplementary Fig. 1). Thus, the PB-derived ANAs represented serological anti-nuclear reactivity well and were closely associated with SLE disease activity.

**Table 1:** Reactivity of the ANA clones isolated from acute SLE patients.

Clone ID	Patient ID	IFA (Hep2)		ELISA^c^								
		Pattern^a^	Conc.^b^	RNP/Sm	SS-A/Ro	SS-B/La	dsDNA	ssDNA	Histone	CL	Insulin	LPS
51B12	SLE5	Sp	2	+++	−	−	−	−	−	−	−	−
51C5	SLE5	Sp	2	+++	−	−	−	−	−	−	−	−
51F1	SLE5	Sp	0.67	+++	−	−	−	−	−	−	−	−
91E12	SLE9	Sp	0.67	+++	−	−	−	−	−	−	−	−
113F3	SLE11	Sp	0.67	+++	−	−	−	−	−	−	−	−
71G1	SLE7	Nucl	2	−	−	−	−	−	+	+	−	+
71F12	SLE7	Ho	**≦**0.2	−	−	−	+++	+++	−	−	−	−
72H11	SLE7	Ho	**≦**0.2	+	−	−	+	+	+	++	+	+
74F4	SLE7	Ho	**≦**0.2	−	−	−	−	−	−	−	−	−
74G9	SLE7	Ho	**≦**0.2	−	−	−	−	−	−	−	−	−
74H4	SLE7	Ho	**≦**0.2	−	−	−	+	+	+	+	+	−
10C3	SLE10	Ho	**≦**0.2	−	−	−	−	−	−	−	−	
10C6	SLE10	Ho	2	+	−	−	+	+	+	+	+	+
121G9	SLE12	Ho	**≦**0.2	−	−	−	+++	+++	+	+	−	+

^a^Sp: speckled, Nucl: nucleolar, Ho: Homogenous.

^b^The lowest reactive concentration in IFA (μg/ml).

^c^+++positive at <0.0313 μg/ml, ++positive at 0.0313–0.0625 μg/ml, +positive at 0.0625–0.25 μg/ml, −negative at 0.25 μg/ml.

The specificities of the ANA clones were evaluated in ELISA (Table 1). All ANAs that show speckled staining were specific to RNP/Sm. 71F12 and 121G9, which showed homogenous anti-nuclear staining, showed strong and nearly exclusive reactivity toward both ds- and single-stranded DNA (ssDNA). Five clones showed weak polyreactivity. Albeit 74F4, 74G9 and 10C3 showed typical homogenous nuclear staining even at low concentrations, it did not react with any tested nuclear antigens in ELISA.

### Somatic mutation contributes highly to the self-reactivity of ANAs

**Figure 2:**
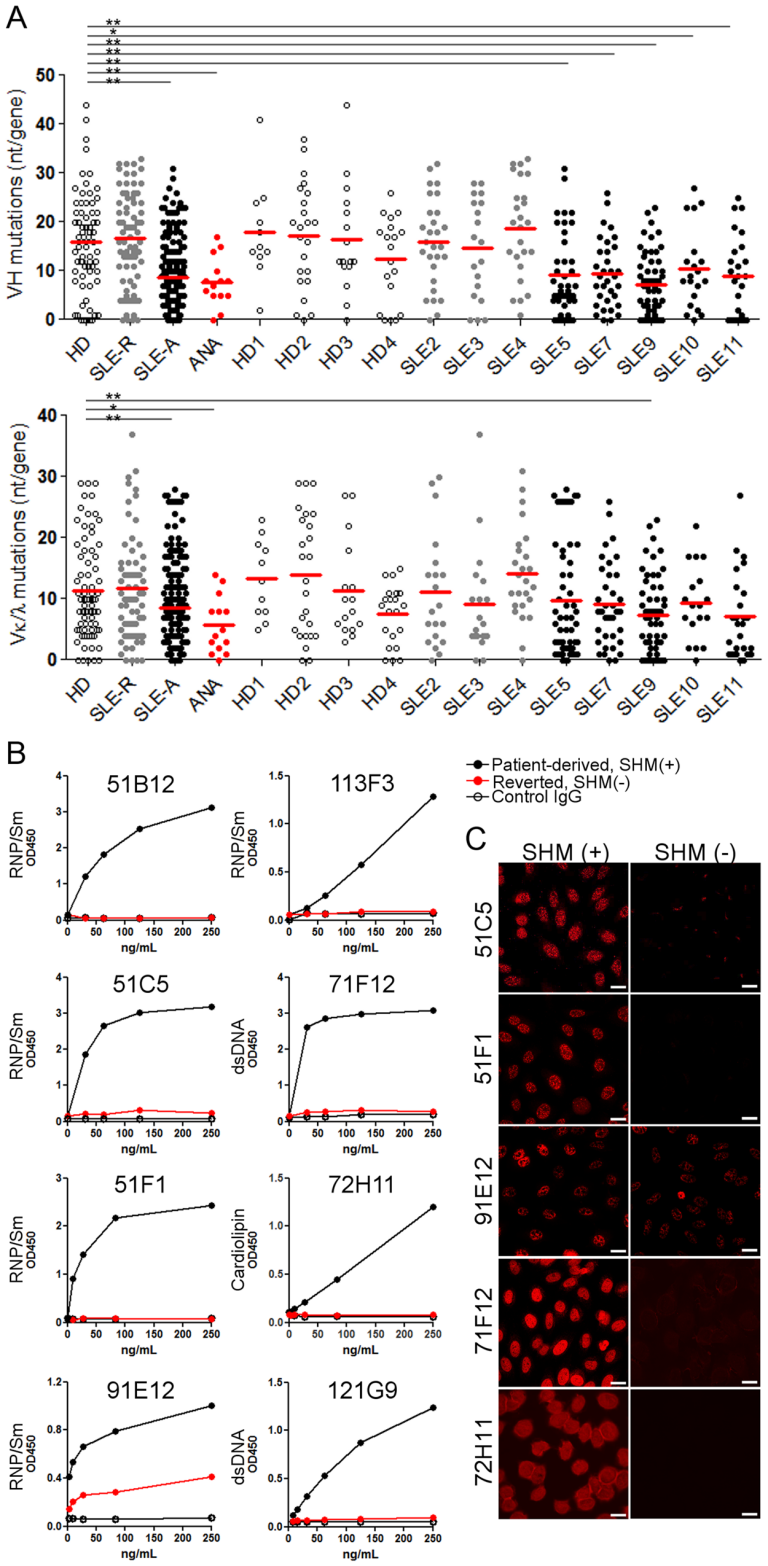
A low frequency of somatic mutations largely contributes to self-reactivity of the isolated ANAs. (**A**) V mutations (nt) of the mAbs from healthy donors (HD, 3 donors, n = 69), SLE remission (SLE-R, 3 donors, n = 72), and acute patients (SLE-A, 5 donors, n = 145), and the ANA clones (n = 14). Mann-Whitney test (*P ≦ 0.05, **P ≦ 0.005). (**B**,**C**) Reversion assay in ELISA (**B**) and IFA (**C**). The reactivity of patient-derived mutated (black: SHM [+]) or reverted (red: SHM [−]) antibodies in ELISA for the respective self-antigens are shown.

The immunoglobulin (Ig) variable sequences from acute SLE patient-derived antibodies contained significantly fewer somatic mutations (Fig. 2A; SLE-A: mean VH and VL nucleotide [nt] mutations/gene were 8.78 and 8.51, respectively) than those from SLE patients in remission (SLE-R VH: 16.6 and VL: 11.72) and healthy donors (HD VH: 15.9 and VL: 11.4). The rate of V mutations was also lower in the identified ANA clones (Fig. 2A; ANA VH: 7.79 [min. = 0; max. = 17] and VL: 5.79 [min. = 0; max. = 14]), and one clone (74H4) had no somatic mutation. Because unmutated germline (GL) antibodies of selected ANA clones showed no reactivity or reduced self-reactivity in ELISA and anti-nuclear reactivity in IFA (Fig. 2B), the self-reactivity of most ANAs was dependent on somatically mutated residues, although the numbers of VH and VL mutations of these clones were lower than those of mAbs derived from patients in remission and healthy donors. The only exception appeared to be 74H4, which had no mutation, but showed strong anti-nuclear reactivity.

### Clonal size and selection signature of ANAs in SLE

**Figure 3:**
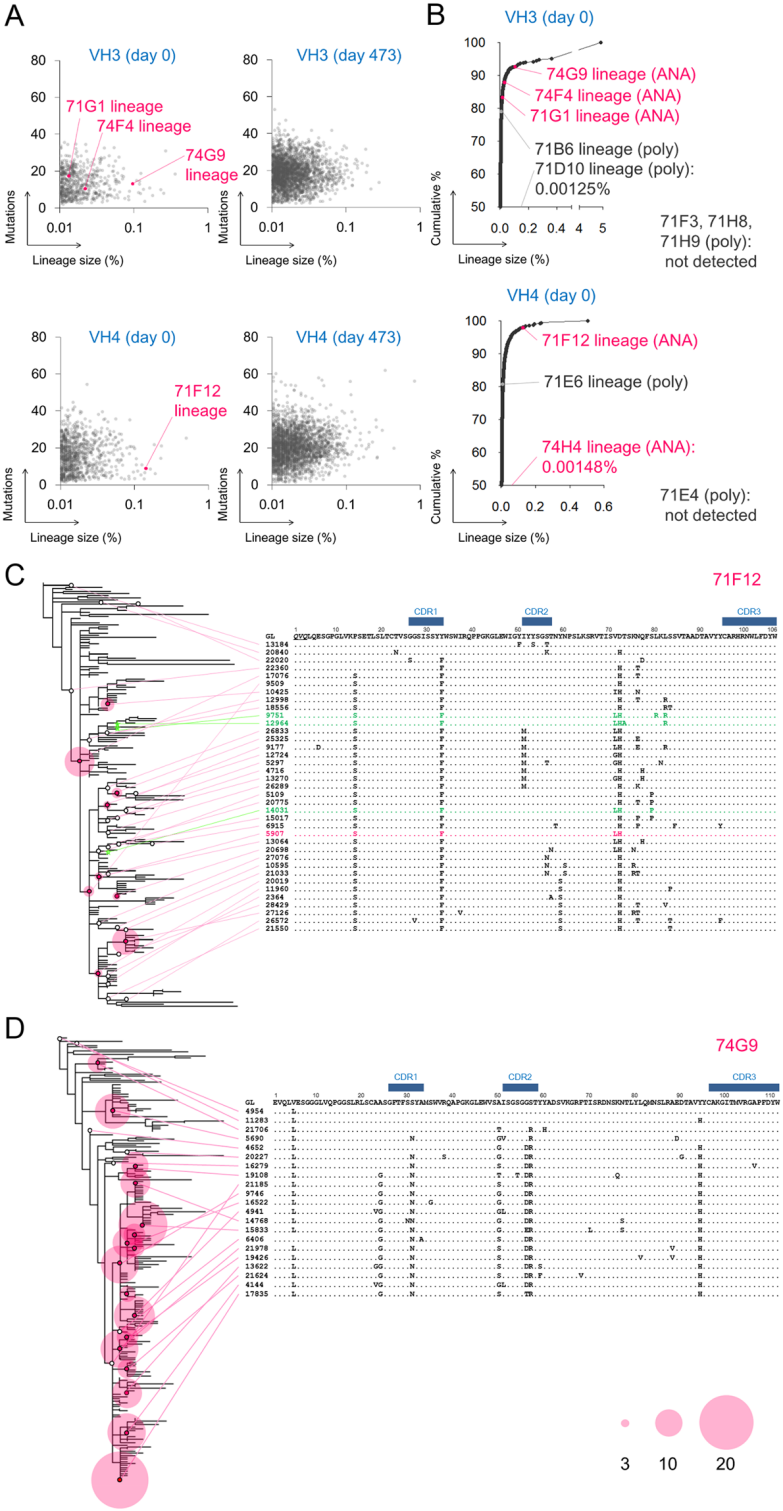
The ANA lineages undergo clonal diversification in acute SLE. (**A**,**B**) Mutations and sizes and cumulative distribution of the VH3 and VH4 lineages from the acute subject, SLE7. The ANA and non-ANA polyreactive lineages are indicated. (**C** and **D**) Phylogram of the 71F12 (**C**) and the 74G9 ANA lineage (**D**). Representative sequences are shown. Red circles reflect the number of identical reads (≧3). The primer-derived sequences are underlined. The identical sequence to the originally isolated 71F12 is colored in red. Sequences found in the subject at day 154 are shown in green.

To gain insight into the distribution and evolution of ANA clonal lineages in SLE, by using HTS, we analyzed the Ig heavy chain repertoire of one of the patients, SLE7 before (day 0) and during treatment (at days 154 and 473). We amplified and sequenced transcripts of the VH3 and VH4 families from total blood lymphocytes, which would cover a majority of human Ig repertoires including several isolated ANA clones of this patient. This approach enables us to quantify the sizes and intraclonal diversification of ANA clone lineages at transcript level, which reflects their contribution to the serum self-reactivity. In total, 87,109 VH3 clonal lineages (240,661 unique sequences) and 52,954 VH4 lineages (134,793 unique sequences) were obtained (Fig. 3A,B, and Supplementary Table 3). It appeared that B cells in the acute SLE patient were rather polyclonal. Sizes of dominant lineages in SLE7 were much smaller than those in donors who were vaccinated (FV) for, or infected with influenza virus (FI), where antigen-specific, oligoclonal response was expected (Supplementary Fig. 1 and Supplementary Table 4). For example, in the VH4 transcripts, sum of the top 12.9% lineages in SLE7 accounted for 50% of the total sequences, whereas only 1.4% and 1.2% from the top did so in FV and FI, respectively.

The isolated ANA sequences were still found in large clonal lineages. The ANA 71F12, an anti-DNA antibody belonged to the 6th largest lineage in VH4 sequences (Fig. 3B and Supplementary Table 3). Three VH3 ANA lineages, 74G9, 74F4 and 71G1, were also found in the HTS and ranked 16th, 176th and 391st, respectively (Fig. 3B and Supplementary Table 3). Exceptionally, sequences related to 74H4 that had no somatic mutation were barely detected (8 total sequences). The ANA lineages rapidly shrank or disappeared in sequences of the subjects after clinical treatment (Supplementary Table 3) in line with the diminished symptoms of SLE7 after treatment (data not shown).

In contrast to the ANA lineages, only 3 out of 7 non-ANA polyreactive lineages encoded by VH3 or VH4 were found in the HTS data (Fig. 3B). Detected polyreactive lineages were smaller than the ANA lineages: 71B6, 71D10 (835th and 15,216th in the VH3 lineages, respectively) and 71E6 (278th in the VH4 lineages) (Fig. 3B, and Supplementary Table 3). Therefore, ANA clones appear to have a greater ability to expand compared to polyreactive clones in the acute phase of SLE.

The phylogenetic analysis of the ANA lineages delineated the evolution of self-reactive B cells in SLE. In the anti-DNA 71F12 lineage, a majority of the diverse sequences shared three amino acid mutations, one in HCDR1 (Y33F) and two in the framework regions (P14S and D72H) (Fig. 3C). Three sequences found in the subject at day 154 were divergent from the 71F12 sequence, but still shared these three mutations (Fig. 3C; colored in green). Similarly, the ANA 74G9, 74F4 and 71G1-related sequences earned a range of somatic mutations, some of which were conserved well in each lineage (Fig. 3D and Supplementary Fig. 1). These results indicated that the ANA clones underwent clonal diversification and selection in the acute SLE patient.

### High-affinity anti-DNA antibodies isolated from acute SLE subjects are capable to bind to both ds- and ssDNA

**Figure 4:**
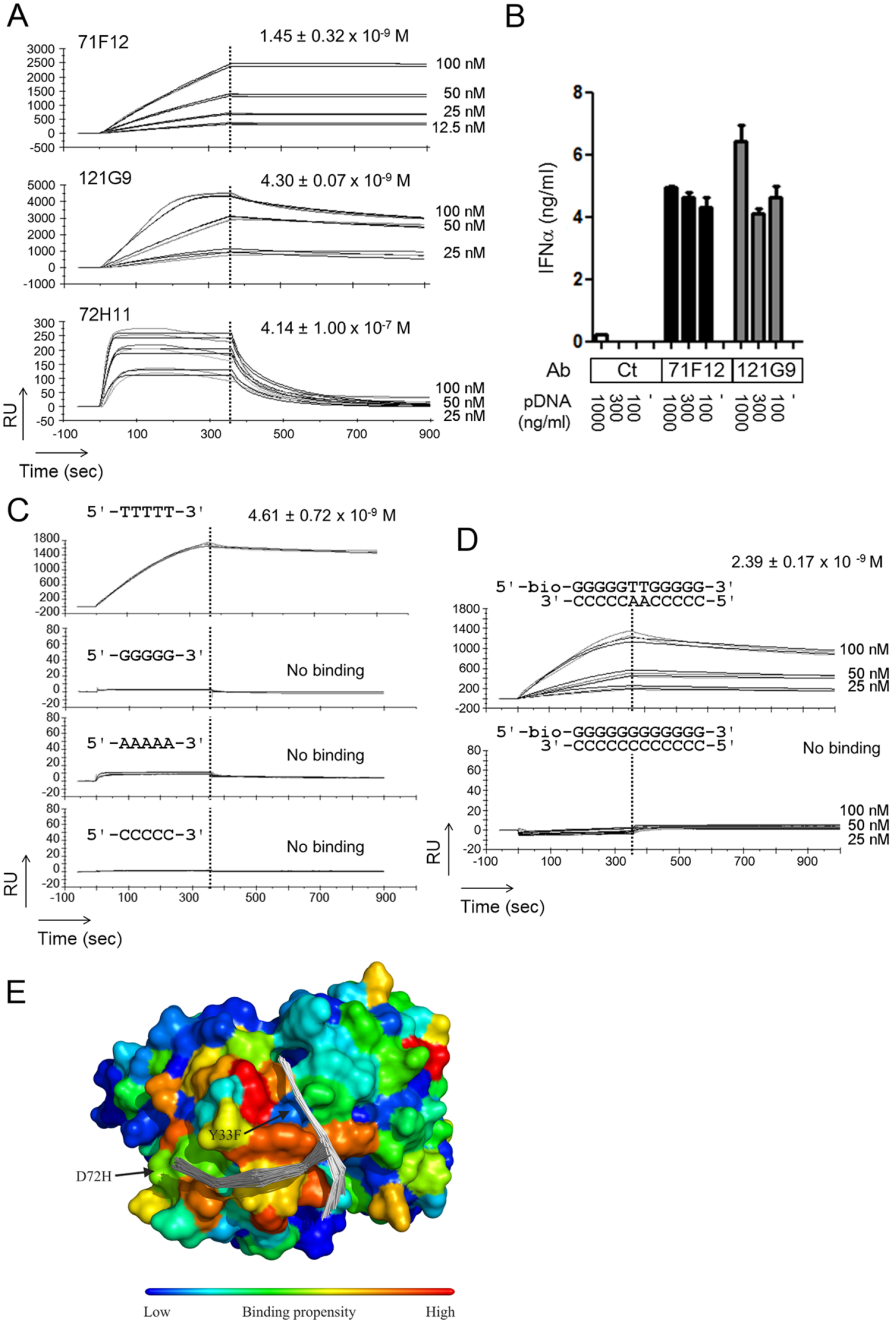
High-affinity anti-DNA antibodies bind to both ds and ssDNA, and sensitize PBMC to dsDNA for IFNα production. (**A**) SPR analysis of 71F12 and 121G9, and polyreactive clone, 72H11. Biotinylated dsDNA (5′-TAATACGACTCACTATAGGG -3′) was immobilized on the chip. (**B**) Enhanced IFNα production of PBMC by 71F12 and 121G9. Freshly isolated PBMCs (5 × 10^5^) were cultured with different amounts of pDNA (100, 300, or 1,000 ng/ml) and 5 μg/ml of anti-DNA antibodies, or control human IgG_1_ (Ct). IFNα concentration was determined by ELISA. (**C** and **D**) 71F12 preferentially binds to thymine-containing DNA. 71F12 (100 nM, unless otherwise indicated) was loaded onto the sensor chip immobilized with ss (**C**), or dsDNA (**D**). The mean values of K_D_ ± s.e.m. from three experiments are shown. (**E**) Flexible docking of ssDNA to 71F12 antibody. Two 5-mer ssDNA binding sites are indicated by backbone traces of the top-100 solutions within the top two clusters. The sites predicted to have high DNA binding propensity are indicated by warmer colors mapped onto the molecular surface of the antibody. Two of the mutations from the germline ancestor (Y33F and D72H) are indicated.

Among the ANAs we isolated, clones 71F12 and 121G9 were highly reactive to both ds and ssDNA. The surface plasmon resonance (SPR) binding experiments revealed that they bind to oligo dsDNA with nanomolar dissociation constants (Fig. 4A), which were much smaller than the values of polyreactive 72H11 (equilibrium dissociation constant [K_D_] = ~400 nM) (Fig. 4A). This type of anti-DNA antibodies may represent IgG antibodies with anti-ds and ssDNA reactivity, which is shown to be eluted from the kidneys of the disease model mouse and SLE patients^15,16^. In addition, both 71F12 and 121G9 facilitated interferon α (IFNα) production of peripheral blood mononuclear cells (PBMCs) in the presence of low concentration of plasmid DNA (pDNA) (Fig. 4B), as previously reported for SLE sera with anti-DNA reactivity^17^, suggesting their pathogenic potency. It should be noted that 71F12 has base specificity, as this clone exclusively bound to the thymine-containing ssDNA and dsDNA (Fig. 4C,D). Flexible docking of 5-mer ssDNA to a homology model of 71F12 *in slico* suggested that the ssDNA tended to be situated close to the antibody at positions 33 and 72 (Fig. 4E).

### Structural basis of antigen recognition by the anti-DNA clone 71F12 supports antigen-dependent selection of the ANA lineage

**Figure 5:**
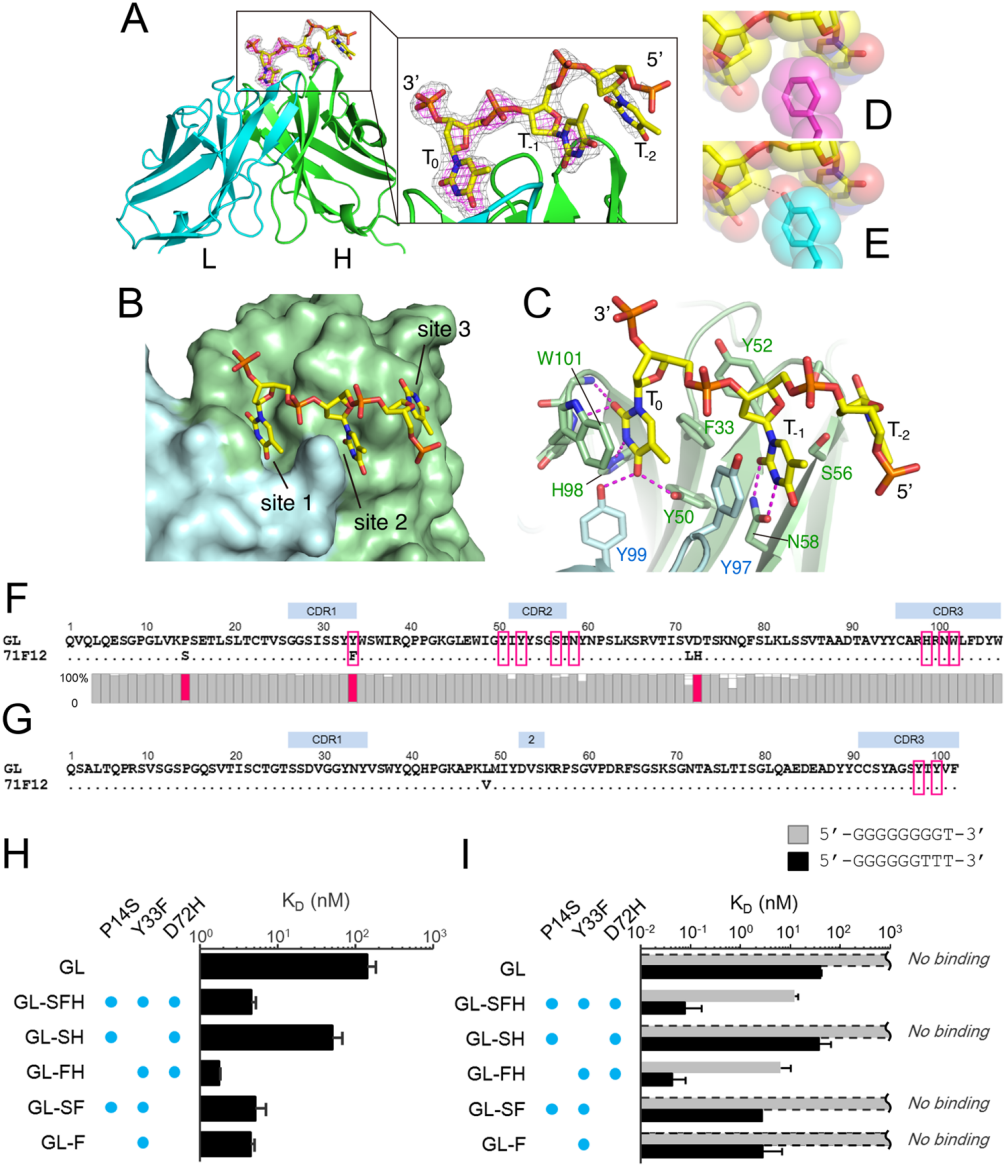
Structural basis of the DNA recognition by 71F12. (**A**–**C**) Structure of the 71F12 Fab-DNA complex determined by crystallography. The DNA-binding sites (sites 1–3, **B**). Thymine bases (T_0_, T_−1_, and T_−2_, **C**). Hydrogen bonds are denoted by dashed magenta lines. (**D** and **E**) Replacement of F33 (**D**: magenta) with Y33 (**E**: cyan) would cause steric hindrance (dashed line). (**F** and **G**) Sequences of the 71F12 heavy chain (**F**) and light chain (**G**). The contacting residues are boxed. For the heavy chain, the conservation of each position from the HTS is shown below as gray bars, except for the three conserved mutations colored in red. (**H** and **I**) The 71F12 IgH-GL antibodies with indicated mutations were tested in SPR for the interaction with 5′-TTTTT-3′ (**H**), or 9-mer DNAs with the indicated sequences (**I**). The mean values of K_D_ ± s.e.m. from three experiments are shown.

To clarify the structural basis underlying the DNA recognition by the 71F12 antibody, the crystal structure of the 71F12 fragment antigen binding (Fab)-ssDNA (5′-TTTTT-3′) complex was solved (Table 2). Clear electron density attributable to the bound DNA was visible at the antigen-binding cleft of the Fab molecules (Fig. 5A), which was consistent with the docked models (Fig. 4E). The DNA was mostly recognized by the heavy chain at three anchor points (sites 1–3) each utilizing the thymine base projected toward the antibody (Fig. 5A,B). Electron density was only visible for a trinucleotide segment, which became weaker toward the 5′ direction (Fig. 5A). Therefore, we speculated that the poly-thymine segment could dock onto the 71F12 antibody with different registers while maintaining the prominent thymine recognition at site 1 (Fig. 5B).

**Table 2:** Data collection and refinement statistics.

	Complex	Apo
Data collection		
Space group	*R*3 (*H*3)	*C*2
Unit Cell Parameters	*a* = *b* = 147.5 Å,*c* = 112.8 Å	*a* = 139.4 Å, *b* = 41.2 Å, *c* = 156.0 Å, *β* = 113.1°
Resolution [Å]	50.0–2.10 (2.14–2.10)	50.0–2.05 (2.09–2.05)
No. of unique reflections	53509 (2696)	52059 (2581)
Completeness [%]	100 (100)	99.9 (100)
*R*_sym_ [%]^a^	11.5 (85.7)	8.3 (61.9)
Redundancy	5.8 (5.6)	4.4 (4.3)
*I*/σ(*I*)	23.1 (2.6)	22.8 (2.3)
Refinement		
Resolution range [Å]	26.33–2.10 (2.15–2.10)	29.65–2.05 (2.10–2.05)
Twin law	*k*, *h*, −*l*	—
Twin fraction	0.330	—
No. of atoms		
Proteins	6239	6227
ssDNA	64	0
PO_4_	0	10
Water molecules	156	194
*R*_work_ (%)^b^	16.3 (20.3)	21.7 (26.7)
*R*_free_ (%)^c^	18.5 (23.6)	25.5 (31.0)
RMSD bond length [Å]	0.007	0.010
RMSD bond angle [°]	1.30	1.45

Values in parentheses correspond to the highest resolution shell.

^a^ *R*_sym_ = 100 × Σ|*I*_*hkl*_ − <*I*_*hkl*_> |Σ *I*_*hkl*_, <*I*_*hkl*_> is the mean value of *I*_*hkl*_.

^b^ *R*_work_ = 100 × Σ||*F*_*o*_| − |*F*_*c*_||/Σ|*F*_*o*_|.

^c^ *R*f_ree_ was calculated from the test set (5% of the total data).

The thymine base at site 1 (designated as T_0_) is deeply inserted into a pocket formed by F33, Y50, H98, N100 and W101 of the heavy chain and Y97 and Y99 of the light chain, and is specifically held via numerous hydrogen bonds and stacking interaction with W101 (Fig. 5C). The structure is only compatible with pyrimidine bases at the T_0_ position, because purines cannot be accommodated in the pocket (Supplementary Fig. 2). Thymine specificity is ensured by numerous hydrogen bonds including the bidentate hydrogen bond donation by Y50 (H, the heavy chain) and Y99 (L, the light chain). Cytosine recognition is expected to be weaker because the tyrosines have to function as hydrogen bond acceptor (blue dotted lines, Supplementary Fig. 2), which is known to be less preferred^18^. In site 2, the thymine base at T_−1_ is half exposed, but still makes extensive contacts with the antibody via F33 (H), S56 (H), N58 (H) and Y97 (L), with the critical involvement of double hydrogen bonds with N58 (H) (Fig. 5C). The interaction mediated by site 3 seems to provide minor contributions to the affinity and base specificity, because the T_−2_ base merely stacks onto the convex ridge formed by S54 (H) and S56 (H) at the periphery of the antigen binding site (Fig. 5B,C). Y52 (H) contributes to the interaction by contacting T_0_ and T_−1_ ribose moieties. From the nature of the binding interface described above, we predict that a DNA antigen should contain a sequence of 5′-NTT-3′ to be recognized by 71F12 with the optimal affinity, although a substitution at the second nucleotide position would be tolerated, with a modest reduction in affinity. In fact, the triple mutant antibody (71F12GL-SFH), which has essentially the same binding property as 71F12, recognized a 9-mer oligonucleotide containing one thymine at the 3′ end (5′-GGGGGGGGT-3′), albeit with an about 150-fold reduced affinity than that toward an oligonucleotide containing three thymines at the end (5′-GGGGGGTTT-3′) (Fig. 5I).

The structural analysis above unraveled a striking fact that most of the antibody residues involved in the DNA recognition are not mutated from the GL sequence, except for the F33 in HCDR1 (Fig. 5F,G). The 71F12 GL antibody could bind to 5′-TTTTT-3′ at ~50-fold lower affinity compared to 71F12 in SPR (Fig. 5H). The conservative mutation Y33F in the heavy chain is critical for higher affinity because the hydroxyl group would cause steric impedance with the DNA backbone (Fig. 5D,E). The decisive role of this residue was experimentally confirmed by introducing the single Y33F mutation into the GL antibody, which resulted in the affinity gain toward 5′-TTTTT-3′ (Fig. 5H).

Among the four 71F12 heavy chain residues that underwent somatic mutation, three (P14S, Y33F, and D72H) were highly prevalent within the lineage (Fig. 5F). Although the selection of Y33F mutation can be rationalized as above, the reasons for the other two mutations found in the framework region are still unclear. When we modeled the structure of the GL antibody, D72 was in close proximity with K75 forming an intra-molecular salt bridge to neutralize the surface charge and the charge-reversing D72H mutation would create a positively charged surface patch (Supplementary Fig. 2). We performed molecular dynamics simulations on 71F12 after docking a long (11-mer) ssDNA and found that the extended DNA tail tended to be attracted to the outer loop region, via long-range electrostatic interactions between the negatively charged phosphate backbone of the DNA and the H72-K75 basic diad (Supplementary Fig. 2). As shown in Fig. 5H, the GL antibody with two mutations P14S and Y33F (71F12GL-SF) showed similar affinity toward 5′-TTTTT-3′ with 71F12GL-SFH, indicating that the D72H mutation has no impact on the binding of 5-mer DNA. When we used 9-mer oligonucleotides, however, the effect of the D72H mutation became evident; the affinity of the antibody 71F12GL-SF to 5′-GGGGGGGGT-3′ was no longer detectable, and the affinity toward 5′-GGGGGGTTT-3′ decreased by ~30 fold (Fig. 5I). Therefore, both Y33F and D72H somatic mutations contribute significantly to the acquisition of high affinity toward a stretch of DNA containing at least one thymine.

### SLE-derived high affinity anti-dsDNA mAbs induce destabilization of the DNA duplex

**Figure 6:**
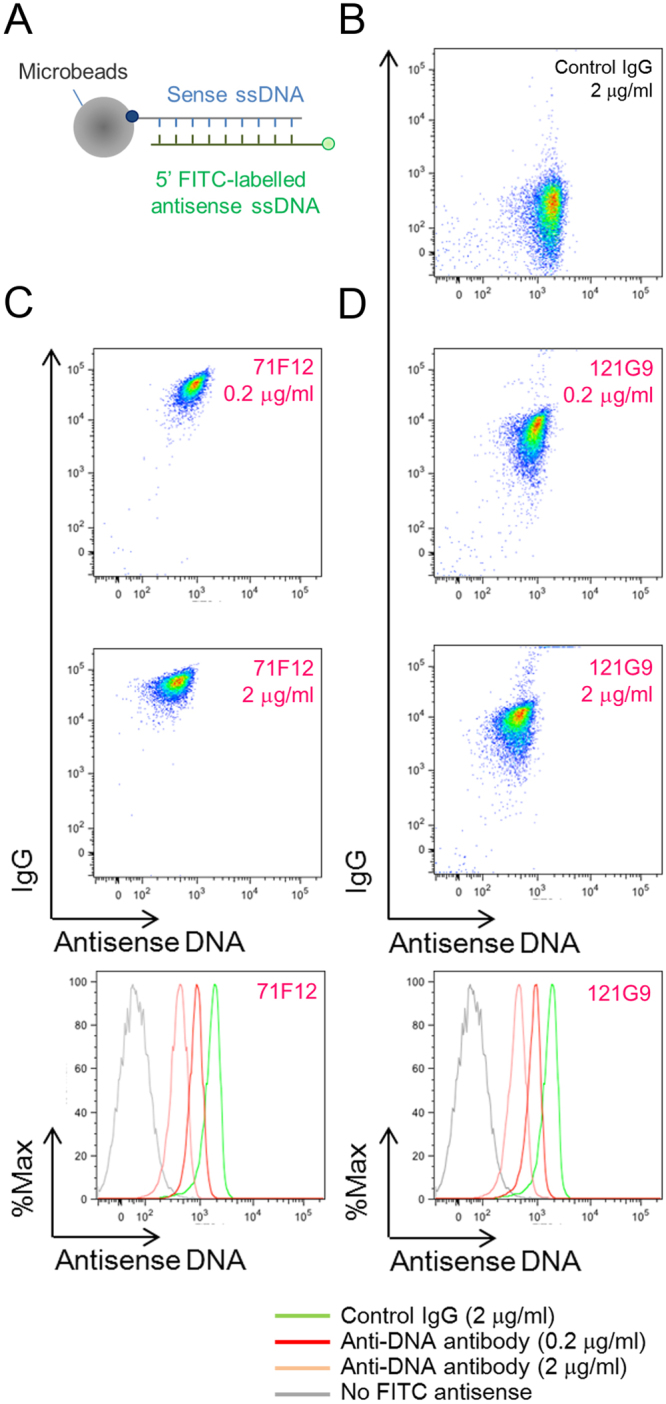
Release of antisense strands of double helices by high-affinity anti-DNA mAbs. (**A**) dsDNA microbead binding assay. Streptavidin-coated microbeads immobilized with double stranded oligonucleotides (5′ biotinylated sense [5′-TATAGTGAGTC-3′] and 5′ FITC-conjugated complementary antisense) were incubated with control human IgG (**B**, 2 μg/ml), 71F12 (**C**), or 121G9 (**D**). Representative results from three independent experiments are shown.

Recognition of dsDNA has long been thought of as a characteristic of lupus-associated autoantibodies. As a segment of dsDNA duplex can undergo spontaneous dissociation under physiological condition^19,20^, 71F12 may selectively bind temporarily formed single stranded segments. To test this hypothesis, microbeads were conjugated with a thymine-containing oligonucleotide, annealed with fluorescent-labeled complementary DNA, and incubated with control antibody or 71F12 (Fig. 6A). As clearly shown in Fig. 6B and C, the fluorescence of the beads-bound complementary DNA was diminished along with the binding of 71F12, indicating that the antibody binding promotes the dissociation of DNA. The same result was obtained when we used another high affinity anti-dsDNA antibody 121G9 (Fig. 6B,D), strongly suggesting that the anti-DNA ANAs in SLE patients that had been considered to be reactive with dsDNA do not necessarily recognize duplexed dsDNA, but can target temporarily exposed single-stranded segments within dsDNA, which may consecutively proceed denaturation and expose more epitopes.

## Discussion

The characteristics and origin of pathogenic autoantibodies in human SLE have not been well defined. This might be attributed to ambiguous definitions of SLE self-reactivity. Self-reactivity and polyreactivity of SLE patients are indistinguishable from those of healthy controls as previously reported for memory B cells^10^ and shown here for blood PBs. On the other hand, we identified a panel of ANAs by IFA appearing only in acute SLE subjects. The isolated ANAs appear to be disease-associated autoantibodies, as they recapitulated nuclear staining patterns by respective patients’ sera at low concentrations. About two thirds of these ANAs were non-polyreactive and highly specific to defined or unidentified nuclear antigens, although the rest showed anti-nuclear reactivity with weak polyreactivity or stickiness in ELISA, which is seen even in some antibodies reactive to certain pathogens^21–23^. Notably, the anti-DNA antibodies we isolated exhibited nanomolar-dissociation constant in SPR, indicating that these clones may represent anti-DNA reactivity in SLE sera. Reversion of mutations in the ANAs to GL residues abolished or significantly reduced their reactivity to respective nuclear antigens and anti-nuclear reactivity, demonstrating a critical role of SHM in SLE self-reactivity.

It has been reported that B cells expressing inherently self-reactive VH4-34 are frequently observed in SLE^13,24–26^. None of our ANAs here were encoded by VH4-34. Although we do not have a clear explanation for this discrepancy, it is possible that this may be due to ethnic or genetic variation, or differences of the disease subtypes. As previously reported^13^, the over-representation of VH4-34^+^ B cells may not be necessarily observed in all SLE patients.

Our HTS analysis used Ig repertoires amplified from PBMC-derived cDNA, which allowed us to seek the ANA lineages from a limited amount of blood. The phylogenetic analysis revealed that in the acute phase of SLE the ANA lineages undergo extensive intraclonal diversification, in which several common mutations were found in each ANA lineage. Taken together with the strict SHM-dependency on self-reactivity of most ANAs, our findings indicate that ANA-producing cells are generated through antigen-driven selection from non-reactive or weakly reactive precursors rather than accidental acquisition of self-reactivity in polyclonal B cell activation. Such ANA clones might be derived through germinal center (GC) reactions in early immune responses, much like in the case of primary response against foreign antigens or pathogens. It is noteworthy that the ANA clone 74H4, which was exceptionally unmutated, was barely detected by the HTS analysis unlike other ANAs carrying critical mutations. This antibody might have been generated by PBs, which underwent an extrafollicular reaction without massive expansion.

In this study, we solved the structure of a high affinity human ANA, which was confirmed to recognize both ss- and dsDNA. To our surprise, the structure revealed that this antibody recognizes thymine-containing oligonucleotides in a manner that is only compatible with ssDNA unlike previously proposed models^5,14^. This apparent discrepancy was reconciled by a subsequent experiments showing that the antibody binding induces concomitant release of a DNA strand, indicating that it can bind to a temporarily formed unpaired segment within a dsDNA and further destabilized a DNA duplex. As another anti-DNA antibody, 121G9 also accelerated the dsDNA dissociation, we speculate that most if not all high affinity anti-dsDNA antibodies reported to represent disease-associated antibodies in SLE patients may be targeted at “loosened” single-stranded segments, which may be present in a released dsDNA upon cell death. Reported co-crystal structures of mouse anti-DNA antibodies with their ligands invariably showed direct recognition of exposed nucleobases by antibodies^27–29^.

Both 71F12 and 121G9 anti-DNA antibodies were capable to enhance IFNα production of human PBMCs in the presence of dsDNA. Anti-DNA antibodies may facilitate incorporation of DNA molecules into cells via Fc receptors^17^. The interaction between endogenous DNA and Toll-like receptor 9 (TLR9) has been shown to be involved in the pathogenesis of SLE in human and mouse^30–32^. Considering that TLR9 binds to ssDNA in the endosomal compartment^33,34^, this type of high affinity anti-DNA antibodies might further contribute to inflammation in SLE by facilitating TLR9 ligation to an agonist through unwinding dsDNA.

The present crystal structure offers a glimpse of how SHM plays a role in the acquisition of self-reactivity over the course of SLE pathogenesis. Although the 71F12 GL antibody possessed detectable affinity toward DNA containing at least three consecutive thymines in the SPR, it did not show any significant dsDNA reactivity in ELISA or nuclear reactivity in IFA, indicating its low affinity. In turn, this suggests that such low affinity-binding may be enough for the B cells to enter to GC reactions. Strikingly, 71F12 gained high affinity against thymine-containing nucleotides by mutating just two residues, one of which lies outside the core antigen binding site. The D72H mutation appeared not only to enhance the binding affinity to long DNA but also to confer broad sequence specificity by reducing thymine dependency, which may contribute to selection of the 71F12 lineage B cells.

In sum, the current study demonstrated, for the first time, the evolutionary process of a human autoantibody by using HTS, which was firmly corroborated by structural analysis. Our results provided genetic and structural evidence that ANA-producing cells are generated through strict antigen-driven selection from non-reactive or less reactive naïve precursor cells probably in GC-like reactions, the presence of which is predicted from previous observations that circulating follicular helper T cells expand in severe SLE^35,36^. These findings will be helpful to understand the immunological etiology and may lead to discovery of novel therapeutic targets for treatment of SLE.

## Methods

### Ethical statement

The consent procedure and the research protocol were approved by Osaka University Research Ethics Committee. All experiments with clinical subjects were conducted in accordance with the approved protocol. All clinical samples were obtained after signed the informed consent at Osaka University Hospital (Osaka, Japan). SLE diagnosis was made based on the American College of Rheumatology (ACR) criteria.

### Single PB sorting and mAb cloning

Sorted single PBs (CD19^lo^ CD138^hi^) were lysed, directly reverse transcribed and then separately amplified by specific PCR for the V regions of IgG, Igκ or Igλ in nested PCR^37^. The products were sub-cloned into mammalian expression vectors, which contain the constant region of human IgG_1_, Igκ or Igλ and the leader sequence derived from mouse Igκ, to be transfected into HEK293T cells cultured in serum-free medium (Thermo Fisher Scientific). Obtained immunoglobulins were sequences and analyzed by IMGT/V-QUEST (www.imgt.org).

### IFA and ELISA

For ANA screening, antibody concentration of each culture supernatant was adjusted to 2 μg/ml or less, was tested in indirect immunofluorescent assay with glass slide of Hep2 cells (Orgentec). Bound antibodies were detected by anti-human IgG-Alexa Fluor 594 antibody (Thermo Fisher Scientific). Recombinant mAbs are tested for self-reactivity in ELISA as described previously^37^. For dsDNA ELISA, linearlized plasmid DNA was used. Bovine insulin, *E*.*coli* lipopolysaccharide (LPS), recombianat hisotone octamer and CL were purchased from Sigma. To remove contaminated DNA, histone was pretreated with DNase I (Takara) at room temperature for 30 min. For RNP/Sm, Sjogren syndrom antigen A (SS-A/Ro) and SS-B/La, the ANA ELISA kit (Orgentec) was used. Serum ANA IgG titer was determined by using ANA HEp-2 kit (Orgentec) according to the manufacture’s protocol.

### IFNα induction by anti-DNA antibody with plasmid DNA

Human PBMCs were seeded on 96-w plate at 5 × 10^5^/100 μl/well to be cultured in complete RPMI1640 medium containing recombinant human IFNγ (400 U/ml, PeproTech) overnight. On the next day, different concentrations of endotoxin-free plasmid DNA (pcDNA3) with or without purified antibodies (5 μg/ml) were added to the culture. After 48-h cultivation, the supernatant was harvested for IFNα ELISA (Human IFNα pan ELISA kit [Mabtech]).

### HTS

The VH region sequences were amplified by PCR with 25 cycles from PBL-derived cDNA. Agarose gel-purified ~400-bp amplicons were subjected to a library preparation. Paired-end sequencing was performed by MiSeq sequencer with the MiSeq 500 kit v2 (Illumina). In the data analysis, the raw sequences were merged using PEAR 0.9.6^38^ after removal of low quality sequences. For analysis of immunoglobulin genes, productive cDNA sequences of longer than 300 bp and average quality score more than 20 are processed. IgBLAST was utilized for the gene assignment of the HTS data. Blastp was used for the comparison and grouped HCDR3 amino acid sequences if the similarity score calculated by similarity matrix was above the certain threshold. The threshold was set by manual investigation of SHMs appeared in a clone and was proportional to HCDR3 length. Sequences belong to same V gene and HCDR3 are considered to be related. Phylogenetic analysis of unique sequences in representative lineages was performed by Mega6^39^ with the Maximal Likelihood method.

### SPR

All SPR data were collected by Biacore T200 (GE) as described elsewhere^40,41^. The Biacore evaluation software (GE) generated K_D_ values by fitting the data to interaction models.

### Docking simulation

For the 71F12-DNA interaction, coarse-grained molecular dynamics (CGMD) was used to flexibly dock DNA molecules to the antibody surface using KOTAI-Dock with the ESPResSo CGMD engine^42^. The DNA-binding propensity at each amino acid position, as described previously for RNA^43^, was used as a contact potential to select realistic DNA binding conformations. For each model, 100 DNA molecules were randomly distributed around the protein to initialize simulations in parallel. The sampling was sufficient to guarantee binding convergence of DNA molecules from different initial conformations.

### Crystallization of 71F12 Fab

Crystals of 71F12Fab-dT_5_ complex were grown from a drop consisting of equal volumes of the protein solution and the reservoir solution containing 0.1 M Tris-HCl (pH 7.0), 0.2 M NaCl, and 1 M sodium citrate. Diffraction data of both structures were collected at the National Synchrotron Radiation Research Center, Taiwan.

### X-ray diffraction experiment, structure determination, and refinement

All data were processed and scaled using the HKL2000 program^44^. Initial phase of 71F12Fab-dT_5_ structure was determined as a single crystal by molecular replacement analysis with PHASER^45^ from the CCP4 packages^46^ using four human antibody Fab structures (Protein data bank [PDB] Id: 4QHK, 4Y5Y, 4IDJ, and 4LLW for V_H_, V_L_, CH1, and CL, respectively) as search models. The initial phase was improved by density modification^47^, and then automated model building was performed with Buccaneer^48^. The structural models were modified with COOT software^49^ with model refinement cycle with REFMAC5^50^. Twin refinement was performed for 71F12Fab-dT_5_ structure.

### Molecular dynamics simulations

Topology file generation followed by addition of 7-bp ssDNA in the crystal structure was done by the tleap program in AMBERTools14^51^ with the AMBER ff14SB force field. The topology file was converted to the Gromacs format by acpype^52^. The complex was solvated into a 10 Å transferable intermolecular potential with 3 points (TIP3P) water box. Ions were added to neutralize the system. Gromacs 4.6.5^53^ was utilized for all calculations described below. The particle Ewald method was used for calculation of the electrostatic potential. The LINCS^54^ algorithm was utilized to constrain the distance between hydrogens and bonded heavy atoms. The Parrinello-Rahman and v-rescale methods were used for pressure and temperature coupling, respectively. During the production runs, we kept positional restraints of 1,000 kJ/mol^−1^nm^−2^ to heavy atoms of the antibody so we could observe dynamics of DNA on a given antibody structure.

### dsDNA microbead binding assay

Three-molar excess FITC-conjugated oligonucleotides (5′-GACTCACTATA-3′ [Thermo Fisher Scientific]) were incubated with complimentary ones conjugated with biotin at 5′-end and annealed before immobilized. Streptavidin microspheres (1.0-micrometer [Polyscience]) were incubated with annealed oligonucleotides in PBS with 1% BSA. Washed beads were bound with 0.2 or 2 μg/ml of 71F12, 121G9 or control human IgG for 1 h at 22 °C. After wash in PBS with 1% BSA, bound human IgG was detected by incubation with anti-human IgG antibody conjugated with APC (Thermo Fisher Scientific) at 4 °C. Intensity of FITC-labelled antisense oligonucleotides was measured by flow cytometer.

### Statistical analysis

*P* values were calculated by the Man-Whitney U tests (two-tailed) using Prism 5 software (Graphpad). *P* values of less than 0.05 were considered statistically significant.

### Data availability

The crystallographic datasets generated during the current study are available in the PDB repository (https://www.rcsb.org/) under accession codes, 5GKR and 5GKS for 71F12Fab-dT_5_ and 71F12Fab-apo, respectively. The other datasets generated during the current study are available from the corresponding author on reasonable request.

## Electronic supplementary material

Supplementary Information
