# Supplementary material for: Clonal evolution and antigen recognition of anti-nuclear antibodies in acute systemic lupus erythematosus
Source: Sci Rep. 2017 Nov 27;7:16428. doi: 10.1038/s41598-017-16681-y (PMC5703881; doi:10.1038/s41598-017-16681-y)

**Supplemental information**

# Clonal evolution and antigen recognition of anti-nuclear antibodies in acute systemic lupus erythematosus

by Shuhei Sakakibara, Takao Arimori, Kazuo Yamashita, Hideyuki Jinzai, Daisuke Motooka, Shota Nakamura, Songling Li, Kazuya Takeda, Jun Katayama, Marwa Ali El Hussien, Masashi Narazaki, Toshio Tanaka, Daron M. Standley, Junichi Takagi, and Hitoshi Kikutani

**Supplementary Table 1. Patient characteristics**

| | Remission | | | Acute | | | | | |
|---|---|---|---|---|---|---|---|---|---|
| Patient ID | SLE2[a] | SLE3[b] | SLE4[c] | SLE5 | SLE7 | SLE9 | SLE10 | SLE11 | SLE12 |
| Date of blood collection | 2012/7/3 | 2012/7/3 | 2012/7/3 | 2013/7/8 | 2014/2/5 | 2014/3/26 | 2014/7/9 | 2014/9/11 | 2015/12/8 |
| Gender | F | F | F | F | F | F | F | F | M |
| Age (yr) | 31 | 41 | 51 | 20 | 51 | 34 | 64 | 18 | 40 |
| Malar rash | - | - | - | + | - | - | - | + | - |
| Discoid rash | - | - | - | - | + | - | - | + | - |
| Photosensitivity | - | - | - | - | - | - | - | - | + |
| Oral ulcers | - | - | - | - | - | - | - | - | - |
| Arthritis | - | - | - | + | + | - | + | - | + |
| Serositis | - | - | - | - | Pericarditis | Pericarditis Pleuritis | - | - | Pericarditis |
| Renal disorder | - | - | - | + | Class IV | Class IV | - | - | Class II |
| Neurologic disorder | - | - | - | - | - | - | + | - | - |
| Hematologic disorder | - | - | - | + | + | - | + | + | + |
| Immunologic disorders | - | - | - | + | + | + | + | + | + |
| Anti-nuclear antibody | *ND*[d] | *ND*[d] | *ND*[d] | + | + | + | + | + | + |
| Others | Prednisone, 11 mg/day | Prednisone, 5 mg/day | Prednisone, 2 mg/day | - | - | - | Anxiety disorder | - | - |

[a] Diagnosed with SLE in 2010: Discoid rash, arthritis and neurologic symptoms.
[b] Diagnosed in 2007: Lupus nephritis (WHO class II).
[c] Diagnosed in 1993: Lupus nephritis (WHO class I).
[d] Not determined.

**Supplementary Table 2. Ig genes and CDR3 AA sequences of the isolated ANAs**

| Clone ID | Donor | VH | DH | JH | HCDR3 | Vκ/λ | Jκ/λ | LCDR3 |
|---|---|---|---|---|---|---|---|---|
| 51B12 | SLE5 | 4-59 | 5-12 | 4 | ARDGFDDYNLDY | Vκ2-30 | 2 | MQGTHWYT |
| 51C5 | SLE5 | 1-46 | 6-19 | 4 | ATRRGSGWYEGGFDY | Vκ2-28 | 3 | MQALQTPRT |
| 51F1 | SLE5 | 3-15 | 6-19 | 4 | TTDHIAVTGTDY | Vλ3-10 | 1 | YSTDSSDNHGV |
| 91E12 | SLE9 | 5-10 | 2-21 | 4 | AREEVGGGAIDY | Vκ4-1 | 4 | QQYYTTPHT |
| 113F3 | SLE11 | 3-11 | 3-10 | 3 | ARDFLDSAYGSGSFDM | Vλ2-8 | 2/3 | SSYAGSNNLGV |
| 71G1 | SLE7 | 3-23 | 7-27 | 4 | AKNARNWGSYYFDY | Vκ3-20 | 1 | QQYGSSPPT |
| 71F12 | SLE7 | 4-59 | 1-20 | 4 | ARHRNWLFDY | Vλ2-11 | 1 | CSYAGSYTYV |
| 72H11 | SLE7 | 1-69 | 1-7 | 3 | ARALGTTRFSAALGI | Vκ3-20 | 1 | QQYGSSPWT |
| 74F4 | SLE7 | 3-21 | 1-26 | 4 | ARRNSGSRFDY | Vλ2-14 | 2/3 | SSYTSTNTFDGVV |
| 74G9 | SLE7 | 3-23 | 3-10 | 4 | AKGITMVRGAPFDY | Vκ1-5 | 4 | QQYNTYSPA |
| 74H4 | SLE7 | 4-59 | 3-10 | 3 | ARRGWRSRSYYAFDI | Vκ3-20 | 3 | QQYGSSLFT |
| 10C3 | SLE10 | 2-5 | 6-13 | 2 | AHRPSGQQLGYWYFDL | Vκ3-20 | 4 | QQYRSSPGLT |
| 10C6 | SLE10 | 4-39 | 3-10 | 4 | ASGRANSYLRPFDY | Vκ3-20 | 1 | QQYGRSPWT |
| 121G9 | SLE12 | 4-39 | 3-16 | 4 | ARGVWGNYRYFDY | Vκ1-39 | 2 | QQSYSPPLT |

## Supplementary Table 3. Summary of the HTS analysis of SLE7

| Sample ID | SLE7 (VH3, Day 0) | SLE7 (VH3, Day 154) | SLE7 (VH3, Day 473) | SLE7 (VH4, Day 0) | SLE7 (VH4, Day 154) | SLE7 (VH4, Day 473) |
|---|---|---|---|---|---|---|
| Cell no. (PBMC) | 1,000,000 | 2,000,000 | 2,000,000 | 1,000,000 | 2,000,000 | 2,000,000 |
| Primers | VH3-JH | VH3-JH | VH3-JH | VH4-JH | VH4-JH | VH4-JH |
| Total read no. | 463,981 | 465,684 | 434,731 | 242,704 | 252,560 | 288,164 |
| Unique sequence no. | 240,661 | 158,279 | 174,930 | 134,793 | 100,968 | 105,942 |
| Clonal lineage no. | 87,109 | 25,518 | 45,215 | 52,954 | 31,807 | 25,562 |
| 71G1 ln (ANA, VH3) | 32 (0.0133%) | 0 | 0 | - | - | - |
| 74F4 ln (ANA, VH3) | 53 (0.0220%) | 0 | 0 | - | - | - |
| 74G9 ln (ANA, VH3) | 233 (0.0968%) | 0 | 0 | - | - | - |
| 71B6 ln (Polyreactive, VH3) | 20 (0.00831%) | 0 | 0 | - | - | - |
| 71D10 ln (Polyreactive, VH3) | 3 (0.00125%) | 0 | 0 | - | - | - |
| 71F12 ln (ANA, VH4) | - | - | - | 221 (0.164%) | 3 (0.00297%) | 0 |
| 74H4 ln (ANA, VH4) | - | - | - | 2 (0.00148%) | 0 | 0 |
| 71E6 ln (Polyreactive, VH4) | - | - | - | 26 (0.0193%) | 0 | 0 |
| 71E4 ln (polyreactive, VH4) | - | - | - | 0 | 0 | 0 |

## Supplementary Table 4. Summary of the HTS analysis of FV and FI

| Sample ID | FV (VH3) | FV (VH4) | FI (VH3) | FI (VH4) |
|---|---|---|---|---|
| Cell no. (PBMC) | 1,000,000 | 1,000,000 | 1,000,000 | 1,000,000 |
| Primers | VH3-JH | VH4-JH | VH3-JH | VH4-JH |
| Unique sequence no. | 149,985 | 133,354 | 133,425 | 125,412 |
| Clonal lineage no. | 49,255 | 27,289 | 43,463 | 26,983 |

**Supplementary Figure legends**

**Supplementary Fig. 1. ANA isolation and HTS analysis.** (A) Sorting strategy for single cell antibody cloning. Blood PBs (7-AAD$^-$ CD19$^{low}$ CD138$^+$) were sorted from freshly isolated PBMCs. (B) IFA on Hep2 cells with ANA clones isolated from acute SLE patients. Unrelated human IgG was used as negative control. Representative results are shown. Bars = 20 μm. (C) Serum ANA titers. Serum ANA titer was determined by IFA with serial diluted sera. (D) VH3 and VH4 Ig repertoires derived from a donor who received a multivalent influenza HA vaccine 7 days before collecting blood (FV) and a convalescent donor who was infected with influenza virus (FI). (E) Phyogenetic analysis of the 71G1 and 74F4 ANA lineages (day 0). The identical sequence to the originally isolated 71G1 is colored in red (#3297). The lineage sequences were analyzed by Maximum-likelihood method. Red circles represent the number of reads (≧ 3).

**Supplementary Fig. 2. Structural basis of antigen recognition by 71F12.** (A) Binding models of various bases. The models in which a cytosine, adenine, or guanine base is bound at site 1 were built by replacing the thymine base in the crystal structure with other bases without changing other atomic coordination. (B) *in silico* model of the unmutated germline (GL) precursor (upper and bottom left). The D72H mutation of the 71F12 heavy chain disrupts the intramolecular interaction with K75, instead generates the H72-K75 diad (right). (C) Predicted structure model of a longer DNA-71F12 Fab interaction. 3'-end of ssDNA could attach to the outer loop of 71F12 H chain.

# Supplementary Figure 1

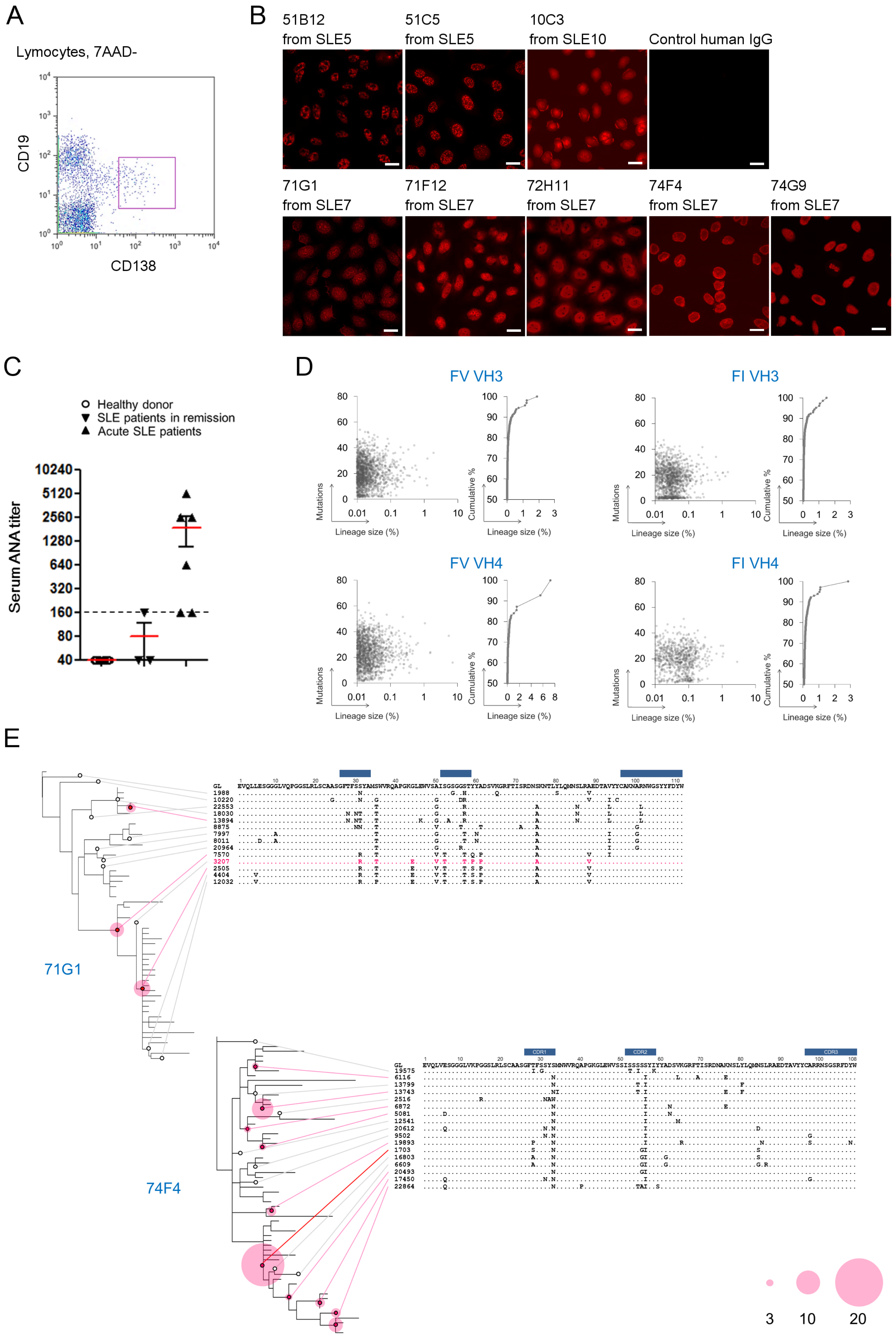

Supplementary Figure 2

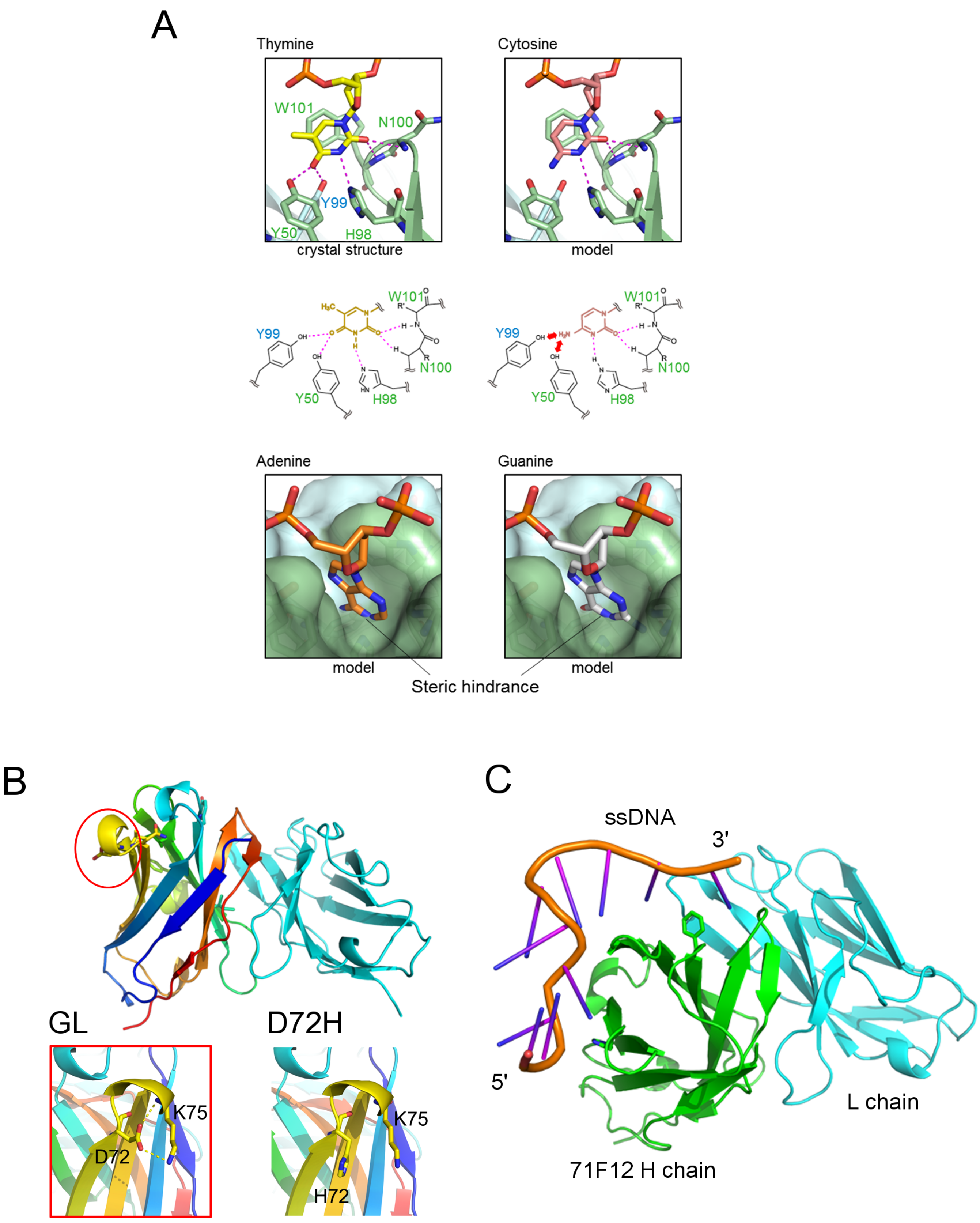

Supplement: Supplementary file 1 — Supplementary Information [file 41598_2017_16681_MOESM1_ESM.pdf]
